# Image rectangling network based on reparameterized transformer and assisted learning

**DOI:** 10.1038/s41598-024-56589-y

**Published:** 2024-03-24

**Authors:** Lichun Yang, Bin Tian, Tianyin Zhang, Jiu Yong, Jianwu Dang

**Affiliations:** 1 Key Laboratory of Optoelectronic Technology and Intelligent Control, Ministry of Education, Lanzhou Jiaotong University, Gansu, Lanzhou China; 2 College of Electronics and Information Engineering, Lanzhou Jiaotong University, Gansu, Lanzhou China; 3 The National Virtual Simulation Experiment Teaching Center of Railway Traffic Information and Control, LanzhouJiaotong University, Lanzhou, 730070 China

**Keywords:** Image rectangling, Single wrap, Re-parameterization, Assisted learning, Electrical and electronic engineering, Computer science, Information technology

## Abstract

Stitched images can offer a broader field of view, but their boundaries can be irregular and unpleasant. To address this issue, current methods for rectangling images start by distorting local grids multiple times to obtain rectangular images with regular boundaries. However, these methods can result in content distortion and missing boundary information. We have developed an image rectangling solution using the reparameterized transformer structure, focusing on single distortion. Additionally, we have designed an assisted learning network to aid in the process of the image rectangling network. To improve the network’s parallel efficiency, we have introduced a local thin-plate spline Transform strategy to achieve efficient local deformation. Ultimately, the proposed method achieves state-of-the-art performance in stitched image rectangling with a low number of parameters while maintaining high content fidelity. The code is available at https://github.com/MelodYanglc/TransRectangling.

## Introduction

Image stitching is the process of combining a set of images captured from different viewpoints, which have overlapping regions, into a single image with a wider viewing angle^1^. This technique has a wide range of applications in various fields, including medical imaging, automated driving, virtual reality, motion detection, and tracking^2^. As stitching technology advances, creating wide-angle images is becoming increasingly popular. However, image stitching primarily focuses on aligning overlapping regions, resulting in irregular boundaries for non-overlapping regions. This limitation restricts the scope of application for image stitching technology. A survey has shown that over 99% of the images in the ‘Panorama’ tab on Flickr (flickr.com) have rectangular boundaries^3^. This suggests that most people prefer to accept stitched images with regular boundaries.

To achieve a rectangular image with regular boundaries, the most straightforward method is to crop out the irregular boundaries of the stitched image. However, this approach results in the loss of significant information about the original content. Image complementation techniques^4–9^ can be used to fill missing regions of an image based on information from intact regions. This method performs better when the missing region is small or the texture structure is relatively simple. However, when the missing region is large or the texture structure is highly complex, the resulting filled region may have serious content distortion.Furthermore, it is important to note that the use of image complementation techniques to obtain a rectangling image may result in the inclusion of misleading information. This can be particularly hazardous in fields such as autonomous driving where accuracy is crucial.

Unlike image complementation techniques, image rectangling techniques do not introduce any additional false content information. They distort the stitched image into a rectangling image by grid distortion based only on the original content information. Traditional methods align different images by optimizing global or local warping^10–12^.However, the traditional method^10–12^ achieves stable image rectangling results in a variety of application scenarios though. However, this method only improves the model’s ability to retain linear structures by introducing a linearity-preserving energy term, which can cause problems such as content distortion in scenes containing nonlinear structures such as portraits.In recent years, deep learning has inspired new developments in the field. It enables the acquisition of deeper semantic features and more detailed information with greater robustness.Recent work^3^ introduced deep learning into the field of image rectangling in order to improve performance. This approach has achieved satisfactory results in a variety of scenarios, including those with nonlinear structures, through multilevel grid warping.The image rectangling method based on deep learning^3^ achieves higher content fidelity by using multilevel warping. This is achieved through a cascading warping design that perceives the content information of the image to be warped from the feature level. However, the design of cascaded warping is prone to the cumulative error problem^13–15^, which can negatively impact the network’s performance. Additionally, this design increases the number of network parameters and warping duration. Furthermore, the rectangling image obtained by warping in this method has some content loss and boundary breakage.

In this work, we investigate joint optimized representations of content and boundaries without introducing additional semantic information. Our approach preserves the original content information while maintaining the straight-line state of the image boundary. Therefore, we propose a new network for rectangling, which is based on the ideas of reparameterized Transformer, assisted learning, and parallel twisted optimal design. The proposed image rectangling network is the main contribution of this paper.

- A single-step twisted image rectangling network was designed, which has better content fidelity based on a reparameterized Transformer.
- An assisted learning network has been designed based on content reconstruction to enhance the performance of image rectangling networks. This is achieved by performing content reconstruction on distorted images.
- TPS is introduced for torsion point control instead of the traditional local mesh torsion, resulting in more efficient parallel computation performance.
- The proposed method achieves state-of-the-art performance in rectangling stitched images, with both qualitative and quantitative comparisons showing superior results compared to methods with higher parametric counts and slower run rates.

## Related work

### Image stitching

The aim of image stitching is to align overlapping regions^16^ in order to reduce artefacts in the stitched image. There are two main approaches to image stitching: adaptive warping and seam-based. Adaptive warping is primarily used to find the optimal uni-responsive matrix through feature matching to ensure alignment of the image content^17–19^. For instance, geometric elements such as points, lines, and edges, as well as depth maps and semantic planes, are utilised to assist in feature matching through additional depth consistency^20^ and plane consensus^21^. Seam-based methods are commonly used in image alignment to determine the optimal splice by optimizing the energy loss function^22–25^. This introduces the challenge of optimizing the allocation of labels along the splice.To achieve satisfactory stitching results, Liao and Chen^26^ proposed a new iterative seam estimation method that uses a hybrid quality evaluation method to assess the pixels along the stitched seams. The iteration is considered complete when the seam undergoes a negligible change from the previous one. However, this method may not be effective for large parallax.ChHeng et al.^27^ combined selective consistency loss of seam shape and quality constraints to supervise network learning and ensure the quality of seam stitching. Nie et al.^28^ synthesized seamlessly stitched images through unsupervised learning, using a simple UNET network for seam mask generation, which is prone to problems such as unclear boundaries, discontinuously generated masks, and lack of seam splice metrics and definitions.

Existing image stitching algorithms, although they can reduce projection distortion and maintain the natural appearance of the image, cannot solve the problem of irregular boundaries in the stitched image.

### Image rectangling

The process of image rectangling involves distorting an irregularly bounded stitched image into a rectangular image with regular boundaries. To address this issue, He et al.^10^ proposed a seam carving technique based on the conformal energy function. This technique has a good rectangling effect in scenes with linear structures, but may cause image deformation and content distortion in scenes containing nonlinear structures. Li et al.^11^ proposed a geodesic-preserving energy function that considers image projection. However, the method requires prior knowledge of the projection of the wide-angle image, which greatly limits its application. Zhang et al.^12^ integrated the stitching and rectangling tasks to achieve simultaneous optimisation by using the idea of chunking followed by rectangling. This method directly generates stitched maps with rectangular boundaries. However, the results generated by this method may not always be complete rectangular images, which is not in line with the original intent of rectangling. Nie et al.^3^ proposed a deep learning-based solution that uses progressive residual regression to achieve satisfactory rectangling in multiple scenarios through multilevel mesh warping. This approach comes at the cost of a higher number of network parameters. However, this method places strong constraints on the boundaries of the distorted rectangular image, resulting in some loss of content and mutilation of boundaries.

It is worth mentioning that some fisheye rectangling work^29–31^ is more similar to our stitching rectangling work, but there are some essential differences between the two tasks. Liao et al.^29^ proposed a rectangling task for distortion correction by curve-aware extrapolation, where the corrected image is made to have more regular rectangular boundaries by extrapolating the image content. Shi et al.^30^ proposed an end-to-end fisheye semantic complementation (FSC) approach to transform single-view fisheye images into rectangular images. The approach combines the image complementation task with semantic understanding in fisheye vision. The fisheye rectangling techniques mentioned above are all derived from the restoration task. They add fictitious image information that complements the original image semantically. Liao et al.^31^ proposed a fisheye rectangling technique that optimizes joint content and boundary using a grid twisting process. This preserves the original content while maintaining the straightness of the image boundary. The technique was described in Ref.^31^. However, the subject of the study is different from the rectangling of the stitched image. The input for the study is a wide-view rectangular image with regular but fuzzy boundaries, which is fundamentally different from the stitched image with irregular boundaries.

Existing image rectification algorithms can reduce content distortion, but they cannot simultaneously constrain the boundaries. In this paper, we introduce the idea of re-parameterisation to the image rectification task. We design a lightweight, general rectification network with better content awareness, aiming to reduce the number of parametric quantities and single-level grid distortion.

## Methodology

### Image rectangling baseline comparison

**Figure 1:**
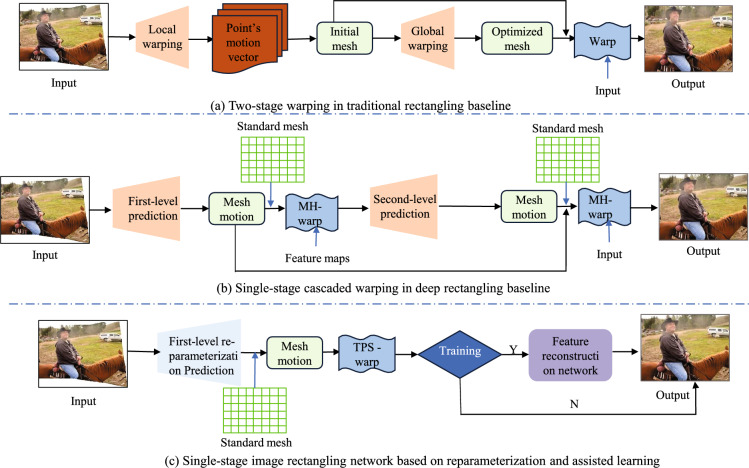
The image in Fig. 1 is from 00014 in the DIR-D test dataset, and the rest of the modules are drawn by visio software. Shows a comparison of image rectangling baselines.The traditional method uses a two-stage twisting process, while the deep learning-based method uses a cascaded network for two twisting processes. The proposed method incorporates a reparameterized strategy to design a lightweight image rectangling network, which requires only one twisting process to achieve the rectangling effect.

Prior to presenting our method, we compare it with existing image rectangling baseline methods to highlight the differences. Figure 1 illustrates the distinctions between the methods.

#### Tranditional baseline

Traditional baseline for rectangling images, such as those described in Refs.^11–13^, typically involve a two-stage process: local warping followed by global warping. In the local warping stage, the initial mesh position is determined through extrapolation and inverse transformation using the seam carving algorithm^32^. The target mesh is then optimised by constraining the conformal energy function in the global warping stage. Finally, the stitched image is warped to generate a rectangular image.Deep Learning Baseline.

#### Deep learning baseline

Deep learning baseline for rectangling images^3^ is a single-stage learning baseline method, unlike traditional methods, the deep learning based method only needs to predict the initial mesh offsets using a residual asymptotic regression strategy. And using a predefined rigid target mesh for efficient parallel computation, it achieves satisfactory rectangling results in a variety of scenarios by means of multi-stage mesh warping.

#### The proposed method

This paper introduces a reparameterized Transformer-based image rectangling network that combines the reparameterized strategy with a TPS grid-point parallel optimisation strategy. Unlike previous work on stitched image rectangling, the proposed method achieves rectangling with only one twisting process. Meanwhile, to achieve higher content fidelity, we design a content reconstruction assisted network to further guide our reparameterised rectangular network for boundary and content tuning, which reduces the burden of complex structure approximation and initialises better starting points for training.

### Method overall

**Figure 2:**
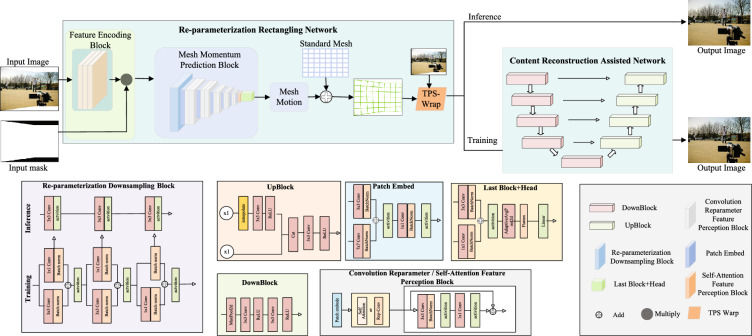
The image in figure is from 00075 in the DIR-D training dataset, and the rest of the modules are drawn by visio software. Overview of the proposed image rectangling network. It takes a stitched image and a mask as inputs. It output the predicted mesh motion. Use tps for distortion to get a corrected stitched image.

Figure 2 illustrates the general framework of rectangling. The re-parameterized image rectangling network takes in a stitched image with irregular boundaries and a masked image. A feature encoding module is used for shallow feature mapping to obtain a more robust representation of the input features. The encoded feature map is multiplied with the mask and then passed to the mesh momentum prediction module for mesh control point momentum prediction. The resulting momenta are summed up with the predefined initial control points. Finally, the image is warped and transformed using the Thin Plate Spline (TPS) transformation strategy^28^ to obtain a warped rectangling image representation. To guide the learning of the re-parameterised image rectangling network, a content reconstruction constraint term is introduced during the training phase. The resulting distorted rectangling image is then passed into the assisted content reconstruction module for processing. During the testing phase, the re-parameterised image rectangling network undergoes parameter integration and directly warps the output rectangling image without passing through the content reconstruction assisted network.

### Network structure

#### Re-parameterization rectangling network

The network comprises a Feature Encoding Block and a Mesh Momentum Prediction Block. The Feature Encoding Block consists of 3 convolutional blocks that perform shallow feature transformations and weaken redundant features on the input image. Simultaneously, the transformed feature map is multiplied by the mask image to incorporate boundary information and improve the subsequent model’s ability to attend to it. The Mesh Momentum Prediction Block comprises 1 Re-parameterization Downsampling Block, 6 Patch Embed Blocks, 10 Convolution Re-parameterization Feature Perception Blocks, and 2 Self-attention Feature Perception Blocks. During model training, multi-branching is employed to enhance feature representations and capture more detailed information. During testing, parallel processing is merged into serial processing, reducing computation and parameters and improving network processing speed. In addition, during mesh warping, we adopted the TPS method^28^ instead of the commonly used spatial transformation method^33^, which eliminates the need to determine the attribution of each to-be-warped pixel to a specific mesh, and has better parallel processing performance.

#### Content reconstruction assisted network

The network comprises of 5 Down-sampling Blocks and 4 Up-sampling Blocks. It performs a detailed representation of the rectangling output image from the previous stage and reconstructs it into a higher content fidelity output representation through a series of convolution, pooling, deconvolution, and jump-joining operations. This guides the re-parameterized rectangling network to learn a better content fidelity representation.

### Loss function

In the rectangling task design, we combine a multinomial loss function to construct a composite loss function to optimise our network. Specifically, grid loss ($L_g$), appearance loss (${L_a}^{L}$), and perceptual loss (${L_p}$) are used in the re-parameterization rectangling stage, and appearance loss (${L_a}^{S}$)and structural similarity loss (${L_{ssim}}$) are used in the content reconstruction assisted stage. This combined loss function is shown in Eqs. (1)–(3):

1 $$\begin{aligned} L_{RR}= & {} L_{g} +{L_a}^{L}+ w_{1} {L_p} \end{aligned}$$

2 $$\begin{aligned} L_{CR}= & {} {L_a}^{s}+ w_{2} {L_{ssim}} \end{aligned}$$

3 $$\begin{aligned} LOSS= & {} \lambda L_{RR}+ {L_{CR}} \end{aligned}$$

where $$L_{RR}$$ is the objective function for the re-parameterization rectangling stage and $$L_{CR} $$ is the objective function for the content reconstruction assisted stage. $$\lambda $$ is the weighting factor of $$L_{RR}$$ , which is used to balance the importance of the two objective functions, here let $$\lambda $$ = 2, $$w_{1} = 0.2$$ and $$w_{2} = 0.2$$.

#### Grid loss

To prevent excessive deformation of the mesh during the twisting phase of the re-parameterized rectangling mesh^3^, mesh constraints are introduced. These constraints include intra-grid losses and inter-grid losses.

The inner grid loss is employed to limit the degree of deformation of the mesh and prevent excessive distortion of its contents. To achieve this, the projected length of the mesh edge on the vertical or horizontal component is calculated using the inner product formula, and it must be greater than a certain threshold. The formula for constraining the mesh edges in the horizontal direction is as follows:

4 $$ L_{h}  = \left\{ {\begin{array}{*{20}l}    {\gamma \frac{w}{n} - {\mathbf{\upsilon }}_{{\mathbf{h}}}  \cdot {\mathbf{i}},} \hfill & {if\;{\mathbf{\upsilon }}_{{\mathbf{h}}}  \cdot {\mathbf{i}} < \gamma \frac{w}{n}} \hfill  \\    {0,} \hfill & {otherwise} \hfill  \\   \end{array} } \right. $$

The given image dimensions are denoted by *h* and *w*, which takes value of 384 and 512. The number of grids in the horizontal or vertical direction of the image is denoted by *m* and *n* , which takes values of 6 and 8. The coefficient $\gamma$ constrains the minimum mesh edge length, and in this case, here let $\gamma$ = 0.125. The horizontal edge vector of the mesh is denoted by $\mathbf {\upsilon _h}$ , and the horizontal unit vector in the right direction is denoted by $\textbf{i}$ . The mesh edge constraints in the vertical direction are shown below:

5 $$ L_{v}  = \left\{ {\begin{array}{*{20}l}    {\gamma \frac{h}{m} - {\mathbf{\upsilon }}_{{\mathbf{v}}}  \cdot {\mathbf{j}},} \hfill & {if\;{\mathbf{\upsilon }}_{{\mathbf{v}}}  \cdot {\mathbf{j}} < \gamma \frac{h}{m}} \hfill  \\    {0,} \hfill & {otherwise} \hfill  \\   \end{array} } \right. $$

$\mathbf {\upsilon _v}$ denotes the vertical edge vector of the mesh and $\textbf{j}$ denotes the vertical unit vector in the downward direction. Integration of horizontal and vertical mesh edge constraints yields the intra-grid loss as shown in Eq. (6):

6 $$\begin{aligned} L_g^{intra}=\frac{1}{(m+1)n}\sum _{\mathbf {\upsilon _h}\in {M_W}} {L_h}+\frac{1}{m(n+1)}\sum _{\mathbf {\upsilon _v}\in {M_W}}{L_v} \end{aligned}$$

Inter-grid losses are employed to limit the overall deformation of the grid, ensuring consistency and enhancing the coherence of the global content information of the rectangular image. To encourage collinearity between two grid edges, calculate the cosine of the angle between two consecutive edges $\mathbf {\upsilon _1}$ and $\mathbf {\upsilon _2}$ in a neighboring grid. where k denotes the number of tuples of two consecutive edges in the grid.

7 $$ L_{g}^{{inter}}  = \frac{1}{k}\sum\limits_{{{\mathbf{\upsilon }}_{1} ,{\mathbf{\upsilon }}_{{\mathbf{2}}}  \in M_{W} }} {\left( {1 - \frac{{{\mathbf{\upsilon }}_{{\mathbf{1}}}  \cdot {\mathbf{\upsilon }}_{{\mathbf{2}}} }}{{\left\| {{\mathbf{\upsilon }}_{{\mathbf{1}}} } \right\| \cdot \left\| {{\mathbf{\upsilon }}_{{\mathbf{2}}} } \right\|}}} \right)}  $$

The final grid loss function is:

8 $$\begin{aligned} L_g=L_g^{intra}+L_g^{inter} \end{aligned}$$

#### Appearance loss

To minimize the sum of absolute differences between the true and predicted values, appearance loss^3^ is utilized. Therefore, both the re-parameterized rectangling grid warping stage and the content-assisted reconstruction stage introduce appearance loss to refer back to the pixel differences between the rectangling image or the content-assisted reconstructed image and the rectangular labels in terms of their appearance content. The calculation is expressed as follows:

9 $$\begin{aligned} L_a^{L}= & {} \Vert I_T - I_{RR} \Vert _1 \end{aligned}$$

10 $$\begin{aligned} L_a^{S}= & {} \Vert I_T - I_{CR} \Vert _1 \end{aligned}$$

where $I_T$ represent the target images, $I_{RR}$ and $I_{CR}$ stand for the output images of re-parameterization rectangling stage and content reconstruction assisted stage individually. The $L_1$ paradigm is denoted by $\Vert \Vert _1$ .

#### Perceived loss

Following the method of Nie et al.^3^, the conv4_2 layer in VGG19^34^ is used to compute the $L_2$ distance between the rectangular labels and the reparameterized rectangling image in the high-level semantic perception. This reduces the feature differences between the re-parameterized rectangling image and the target image by minimising the perceptual loss, as shown in Eq. (11)^35^.

11 $$\begin{aligned} L_p =\Vert \varphi \left( I_T^{L} \right) - \varphi \left( L_{LR} \right) \Vert _2 \end{aligned}$$

where $\varphi \left( \cdot \right)$ denotes the operation of extracting features from the “conv4_2” layer in VGG19, and $\Vert \Vert _2$ denotes the $L_2$ paradigm.

#### Structural similarity loss

The structural similarity (SSIM) loss^36^ is used to measure the structural difference between the predicted image and the target image in terms of brightness, contrast and structural information, which can be applied to the content-assisted reconstruction task. Based on the consideration of the three subtasks performed by the content-assisted reconstruction network, we introduce the SSIM loss to constrain the structural alignment of the output image of the content-assisted reconstruction network with the target image. Its calculation method is shown in Eqs. (12) and (13):

12 $$\begin{aligned} L_{ssim}= & {} 1-SSIM \left( I_T, I_{CR} \right) \end{aligned}$$

13 $$\begin{aligned} SSIM\left( x, y \right)= & {} \frac{\left( 2{\mu _x }{\mu _y }+{C_1}\right) \left( 2{\sigma _{xy} }+{C_2}\right) }{\left( {\mu _x }^2 +{\mu _y }^2+{C_1} \right) \cdot \left( {\sigma _x }^2 +{\sigma _y}^2+{C_2}\right) } \end{aligned}$$

where *x* and *y* stand for $I_T$ and $I_{CR}$, respectively. Here let $C_1={1\times 10}^4$, $C_2={3\times 10}^4$ .

## Experiments

### Experimental environment

#### Implementation details

The model in this paper is based on Pytorch implementation and all experiments were done on a single NVIDIA RTX 2080Ti GPU with 12G of video memory. During training we used a batch size of 7 and 150 iterations, the objective function was optimised using the AdamW optimiser with a pre-set learning rate of $1\times 10^4$. A Warm Up learning rate was used with a warm up step threshold of 10.

#### Dataset and evaluation metrics

The DIR-D dataset^3^ was used for the experiments, which contains 5839 sets of training images and 519 sets of test images, each of which contains an input image with irregular boundaries and an input mask as well as a corresponding target image containing rectangular boundaries.To verify the effectiveness of the proposed method, structural similarity (SSIM), peak signal-to-noise ratio (PSNR) and natural image quality (NIQE) are selected for the quantitative evaluation of the rectangling scheme. Among them, SSIM takes into account the differences in brightness, contrast and structural similarity between the two compared images, which is more in line with human visual characteristics. The closer the SSIM value is to 1, the greater the structural similarity between the two images.PSNR is used to measure the ratio of the maximum signal to the background noise of an image.The higher the PSNR value, the less distorted the image. Natural Image Quality (NIQE) is a reference-free image evaluation metric.The lower the NIQE value, the less distorted the image.

### Results

To demonstrate the adequacy of our work, we perform a qualitative and quantitative comparison with existing image stitching based rectangling methods. Considering the fact that there is less existing work on image rectangling, and to demonstrate the timeliness of the algorithms, we compare it with the fisheye rectangling work related to our work.

#### Quantitative comparison

To further measure the performance difference between the algorithms, we performed a quantitative evaluation and the specific results are shown in Table 1. Table 1 shows the quantitative comparison results of our proposed method with existing image orientation methods.where reference is the result obtained by computing the input irregular image as a baseline. It can be seen that our rectangling method achieves the state-of-the-art rectangling performance with the lowest number of references and the fastest response time for both the referential evaluation metrics (SSIM, PSNR) and the non-referential evaluation metric (NQIE).In comparison, the method of He et al.^10^ , which uses the line-preserving energy function as the optimisation term, focuses on the effect of preserving the linear structure of the original image and neglects the consideration of other non-linearly structured scenes.Although the method of Nie et al.^3^ uses the idea of progressive regression to increase the consideration of scene applicability, its strong constraints on the range of mesh deformations lead to limited content perception in irregular image boundary regions, resulting in more content loss at the boundaries of their rectangling images.The method of Liao et al.^31^ rectangling fisheye images after restoration, but its default input image is a fisheye image with aberrations, so it distorts and deforms the image content of the rich lines, which affects the final output.In addition, it is worth noting that our rectangling method requires only a single level of grid prediction to achieve better rectangles than the multi-level grid prediction used by Nie et al.

**Table 1:** Quantitative comparison on DIR-D, times for each algorithm predicting 11 consecutive rounds on the DIR-D dataset, keeping the average of the results of the last 10 rounds to eliminate errors introduced at the start of the model in round 1.

Methods	#Params(M)	GFLOPs	Time (s)	SSIM$\uparrow$	PSNR$\uparrow$	NIQE$\downarrow$
Reference	–	–	–	0.3245	11.3048	
He et al.’s^10^	–	–	0.4936	0.3775	14.7012	17.15
Nie et al.’s^3^	50.91	35.09	0.0561	0.7141	21.2764	16.75
Liao et al.’s^31^	30.67	19.45	0.0200	0.4861	18.2574	16.78
Our’s	**2.17**	**1.27**	**0.0157**	**0.7420**	**21.4607**	**16.48**

Significant values are in bold.

#### Qualitative comparison

**Figure 3:**
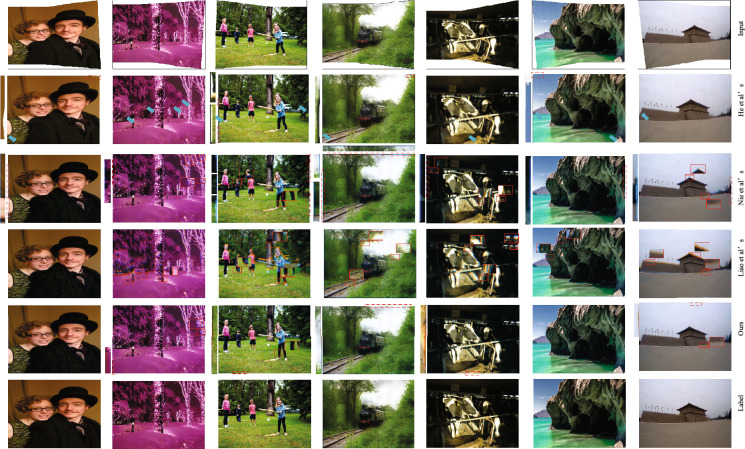
The images in figure are, from left to right, from datasets 00015, 00019, 00024, 00153, 00203, 00209, and 00215 in the DIR-D test dataset,respectively. Plot of the comparison results of the nonlinear scenes for each algorithm. Blue arrows represent distortion failure regions, red rectangular boxes represent twisted and distorted regions, and red dashed boxes represent boundary residual regions.

**Figure 4:**
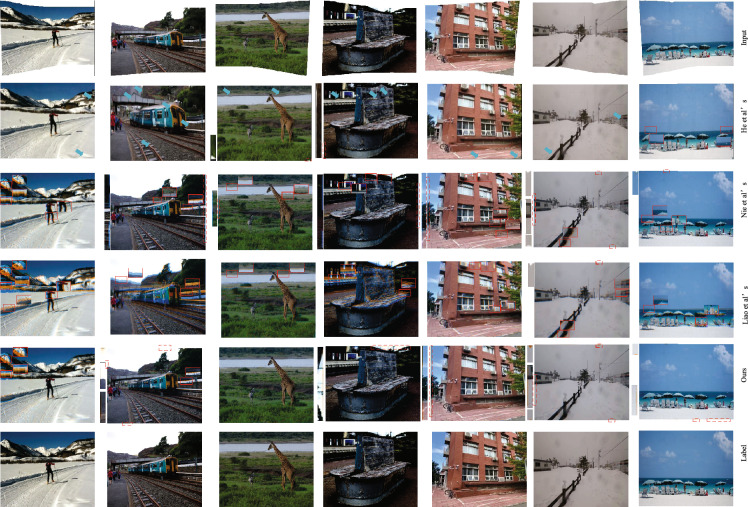
The images in figure are, from left to right, from the DIR-D test dataset 00285, 308, 00334, 00336, 00350, and 00354,respectively. Comparison of the linear structure scene of each algorithm Comparison result map. Blue arrows represent regions with missing content, red rectangular boxes represent twisted and distorted regions, and red dashed boxes represent regions with boundary crippling..

**Figure 5:**
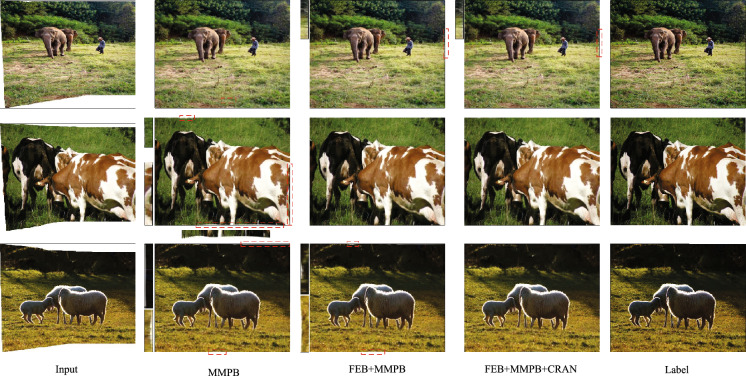
The images in figure are, from top to bottom, from the DIR-D test dataset 00005, 00031, and 00062, respectively. Effect of reparameterized rectangling network, where FEB is the feature enhancement module, MMPB is an acronym for Moving Momentum Prediction Module for Heavy Grids and CRAN for Content Assisted Reconstruction Network. The red dashed box represents the missing boundary region.

Figures 3 and 4 show the rectangling performance of the algorithms for scenes with linear structure and scenes with non-linear structure. In order to facilitate the visual differentiation of the degree of image deformation, we have adopted the method in DAMG^37^ for channel fusion of the rectangling results obtained by Nie and the proposed method with the real labels.If the two are not aligned, blue or orange artefacts appear. As can be seen from Figs. 4 and 5, the method of He et al.^10^ suffers from some content deformation and loss of boundary information in both linear and non-linear structured scenes. The method of Nie et al.^1^ has better shape fidelity than that of He et al.^10^ , but still has a small amount of deformation distortion. The method of Liao et al.^31^ has obvious content distortion (e.g. the proportion of characters in the fourth row and third column is deflated) and some blurring of boundaries. In comparison, the proposed method has better content fidelity.

It can be seen that our rectangling method is superior in terms of content fidelity and rectangling boundary information retention effects. The reasons for this are, on the one hand, thanks to the TPS strategy adopted in the mesh prediction process to predict the movement of the control points, thus controlling the deformation of the non-rigid body and improving the alignment ability of the model in non-linear scenes, and thus the obtained rectangling images have higher content fidelity in non-linear scenes; on the other hand, thanks to the content-assisted reconstruction network guiding the re-parameterized rectangling prediction network, which improves the context awareness of the rectangling network and thus better learning of the grid dynamics.

### Ablation experiment

We have analysed the appropriateness of the proposed method in terms of both the network module and the design of the parallel optimisation strategy. The details are as follows:

#### Network module ablation analysis

Regarding the design of the network module, we mainly consider two aspects: feature encoding and content-based reconstruction support network. Table 2 shows the results of the ablation validation analysis of the network module, and Figure 5 shows the effect diagram of the reparameterized rectangling network. From Table 2, it can be seen that the introduction of the feature encoding module can easily and effectively embed the extracted local semantic information into the high-level feature space, thus improving the scene adaptation of the model^38,39^. The performance of the reparameterized rectangling grid prediction network was improved to a greater extent after the introduction of the content-assisted reconstruction network, and state-of-the-art performance was achieved for both the parametric evaluation metrics (SSIM) and the non-parametric evaluation metrics (NQIE). Therefore, it can be demonstrated that the content-assisted reconstruction network is able to guide the grid prediction task of the re-parameterized rectangling network to some extent. FEB stands for Feature Enhancement Module, MMPB is an acronym for Moving Momentum Prediction Module for Heavy Grids and CRAN for Content Assisted Reconstruction Network. The network effect diagram in Fig. 5 also shows that the Feature Encoding Module improves the ability of the subsequent model to focus on boundary information to some extent.

**Table 2:** Ablation validation analysis for each module of the network.

Strategy			Evaluating metric		
FEB	MMPB	CRAN	SSIM$\uparrow$	PSNR$\uparrow$	NIQE$\downarrow$
	$\checkmark$		0.7171	21.2631	16.5429
$\checkmark$	$\checkmark$		0.7329	**21.4712**	16.4942
$\checkmark$	$\checkmark$	$\checkmark$	**0.7420**	21.4607	**16.4813**

Significant values are in bold.

#### Parallel optimisation strategy analysis

During the design process of the reparameterised rectangular model, to verify the validity of our spatial transformation module, we performed an ablation analysis using the multigrid uni-responsive estimation proposed by Nie et al. as shown in Table 3 below.We have analysed the results in terms of time, parametric evaluation metrics (SSIM, PSNR) and non-parametric evaluation metrics (NQIE), and from the results in the table we can see that the TPS transformations bring an improvement in time, proving that the TPS transformations are effective.

**Table 3:** Comparative analysis of parallel optimisation strategies.

Strategy		Evaluating metric			
MH	TPS	Time(s)	SSIM$\uparrow$	PSNR$\uparrow$	NIQE$\downarrow$
$\checkmark$		0.0847	0.7372	21.4313	16.5743
	$\checkmark$	**0.0133**	**0.7420**	**21.4607**	**16.4813**

Significant values are in bold.

## Conclusion

In this paper, a single-step warping solution for image rectangling is proposed to address the problems of content distortion and missing boundaries in current rectangling methods. Incorporating the ideas of reparameterized and parallel optimal design, a reparameterized transformer-based image rectangling network is designed. Meanwhile, to support the learning of the network, an assisted learning task is constructed to guide the reparameterised rectangling network to learn higher content fidelity lattice momentum representations through content-assisted reconstruction processing. The experimental results show that the proposed method achieves better rectangling than multilevel mesh warping with lower parameter counts and faster run rates. Meanwhile, satisfactory rectangling results are achieved in a variety of application scenarios, and the state-of-the-art rectangling performance is demonstrated in both qualitative and quantitative comparisons with existing rectangling methods.

## Data Availability

The DIR-D sets that support the findings of this study are available at https://drive.google.com/file/d/1KR5DtekPJin3bmQPlTGP4wbM1zFR80ak/view. In addition, all data related to this experiment can be obtained by contacting this email (Jianwu Dang: ylc1377759045@163.com).
